# Identification of three m6A‐related mRNAs signature and risk score for the prognostication of hepatocellular carcinoma

**DOI:** 10.1002/cam4.2833

**Published:** 2020-01-13

**Authors:** Zedong Li, Fazhan Li, Yu Peng, Jianyu Fang, Jun Zhou

**Affiliations:** ^1^ Department of Minimally Invasive Surgery The Second Xiangya Hospital Central South University Changsha Hunan China; ^2^ Department of Gastroenterology The Fifth Affiliated Hospital of Zhengzhou University Zhengzhou Henan China; ^3^ Department of Nursing The Second Xiangya Hospital Central South University Changsha Hunan China

**Keywords:** hepatocellular carcinoma, N6‐methyladenosine (m6A) RNA methylation, nomogram, prognosis

## Abstract

Hepatocellular carcinoma (HCC) is the most common type of liver cancer and is extremely harmful to human health. In recent years, N6‐methyladenosine (m6A) RNA methylation in eukaryotic mRNA has been increasingly implicated in cancer pathogenesis and prognosis. In this study, we downloaded the expression profile and clinical information of 307 patients from The Cancer Genome Atlas database and 64 patients from the Gene Expression Omnibus (GEO) database, and univariate Cox analysis revealed that METTL14 was a prognostic m6A RNA methylation regulator. For further study on the related genes of METTL14, weighted gene co‐expression network analysis was used to find the relationship between METTL14 and gene expression, and univariate Cox analysis and least absolute shrinkage and selection operator (LASSO) methods were used to identify hub genes that may be associated with HCC prognosis. The results indicated that cysteine sulfinic acid decarboxylase, glutamic‐oxaloacetic transaminase 2, and suppressor of cytokine signaling 2 were key genes affecting the prognosis of HCC patients, and m6A methylation of these mRNAs may be regulated by METTL14. Finally, a nomogram was constructed based on the hub gene expression levels, and its prediction accuracy and discriminative ability were measured by the C‐index and a calibration curve. In conclusion, METTL14, an m6A RNA methylation regulator, may participate in the malignant progression of HCC by adjusting the m6A of cysteine sulfinic acid decarboxylase, glutamic‐oxaloacetic transaminase 2, and suppressor of cytokine signaling 2, and these genes are useful for prognostic stratification and treatment strategy development.

## Graphical abstract

In this study, we found that METTL14 may inhibit the progression of hepatocellular carcinoma (HCC) by up‐regulating the expression levels of cysteine sulfinic acid decarboxylase, glutamic‐oxaloacetic transaminase 2, and suppressor of cytokine signaling 2. The main mechanism might be affecting the N6‐methyladenosine process of hub genes. In addition, we established a novel three‐gene signature and nomogram to predict overall survival of HCC, and it might be a useful prognostic and diagnostic classification tool of HCC.

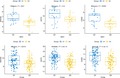

## 1 BACKGROUND

Hepatocellular carcinoma (HCC) is the most common type of primary liver cancer, the sixth most common cancer in the world, and the second leading cause of cancer deaths worldwide.^1^ It is estimated that more than 700 000 people die of this cancer every year.^2^ It is the most common malignant tumor in China and the leading cause of cancer deaths among men.^3^ Hepatitis B virus, hepatitis C virus, and alcohol abuse are the main causes of HCC.^4^ Liver resection and liver transplantation are effective methods for the treatment of liver cancer. However, since most patients are diagnosed at an advanced stage, only 15% of patients are candidates for surgery, and the 5‐year survival rate of potentially curative surgery is only approximately 33%–50%.^5^ Therefore, we need a deeper understanding of the molecular‐level mechanisms causing HCC.

N6‐methyladenosine (m6A) is the most abundant internal modification of mRNA and long noncoding RNA in most eukaryotes.^6^ N6‐methyladenosine plays an important role in regulating mRNA splicing, translation, and stability.^7^ It is methylated on the sixth position of N on adenosine, mainly in the CDS region and 3′ untranslated regions region of the mRNA, affecting mRNA stability, translation efficiency, variable splicing, and localization. m6A mainly modifies the RNA structure by dynamic regulation of "writers" (methyltransferase), "erasers" (demethylase), and "readers" (m6A‐binding proteins that recognize and bind to the m6A site on mRNA).^8^ In "writers" of m6A RNA methylation regulators, studies have shown that methyltransferase like 3 (METTL3) (RNA demethylase 3) and METTL14 (RNA demethylase 14) have 43% identity and are homologues.^9^ In recent years, it has been found that METTL3 can maintain myeloid leukemia through m6A‐dependent translational control^10^ and plays an important role in promoting the translation of the human lung cancer gene.^11^ As a homologue of METTL3, studies have also shown that METTL14 can positively regulate the primary microRNA 126 process in an m6A‐dependent manner to inhibit the metastatic potential of HCC.^12^ These studies have shown that m6A plays an important role in the development of tumors.

In this study, we downloaded the expression profiles and clinical data of The Cancer Genome Atlas (TCGA)‐LICH and http://www.ncbi.nlm.nih.gov/geo/query/acc.cgi?acc=GSE116174. By using univariate Cox analysis methods, we identified prognostic m6A RNA methylation regulators. Furthermore, we determined hub genes that may be regulated by m6A RNA methylation regulators and are associated with HCC prognosis by constructing co‐expression network analysis (WGCNA) and lasso regression analysis (least absolute shrinkage and selection operator [LASSO]); then, based on the combination of selected hub genes, risk‐score models were constructed to evaluate their prognostic applications in HCC. Our results suggest this 3‐gene signature and nomogram might help effectively predict the overall survival (OS) of HCC patients. The close relationship between these hub genes and m6A RNA methylation regulators can also provide new ideas for HCC research.

## 2 PATIENTS AND METHODS

### 2.1 Data collection

The mRNA expression data and corresponding clinical information of patients with HCC were obtained from TCGA cohort and the Gene Expression Omnibus (GEO). This study included the expression profiles of 307 patients with complete follow‐up data in the TCGA database and 64 samples from the http://www.ncbi.nlm.nih.gov/geo/query/acc.cgi?acc=GSE116174 dataset. The TCGAbiolinks package and GEOquery package were used to download the TCGA and GEO data.^13^, ^14^

### 2.2 Identification of prognostic m6A RNA methylation regulators

In this study, we included many m6A methylation regulators, such as “writers”: METTL3, METTL14, WT1‐associated protein, KIAA1429 (also known as VIRMA), RNA‐binding motif 15 (RBM15), and zinc finger CCCH domain‐containing protein 13 (ZC3H13); “readers”: YTH domain‐containing 1 (YTHDC1), YTH domain‐containing 1 (YTHDC2), YTH m6A RNA‐binding protein 1 (YTHDF1), YTH m6A RNA‐binding protein 2 (YTHDF2), and heterogeneous nuclear ribonucleoprotein C (HNRNPC); and “erasers”: fat mass‐ and obesity‐associated protein (FTO) and α‐ketoglutarate‐dependent dioxygenase alkB homolog 5 (ALKBH5). To investigate the differential expression of m6A RNA methylation regulators in tumors and normal tissues, we analyzed the mRNA expression profile of TCGA‐liver hepatocellular carcinoma (including 48 normal samples and 307 tumor samples). Cluster analysis was performed on m6A RNA methylation regulators, and heatmaps and violin maps were presented to show differences. The pheatmap R package and the vioplot R package were used for drawing the plots. Furthermore, for the TCGA data, we used univariate Cox analysis to identify m6A‐related genes associated with HCC prognosis and further validated them using http://www.ncbi.nlm.nih.gov/geo/query/acc.cgi?acc=GSE116174 data (m6A regulatory genes with a *P* value <.05 were considered statistically significant).

### 2.3 Co‐expression network construction and identification of clinically significant modules

The co‐expression network was constructed by the “WGCNA” package in R.^15^ In the TCGA set, genes with variances greater than all variance quartiles were selected, and those genes with larger variances and larger mean variations in different samples were considered. The expression data profile of the selected genes was qualified, and the samples were clustered to detect outliers. Gene clustering modules were identified based on the clinical features (including the expression of the m6A regulatory genes that we selected before) and topological overlap matrix‐based dissimilarity.^16^ Then, the correlations between module eigengenes and clinical traits were calculated to identify the relevant modules. Highly relevant modules were considered significant.

### 2.4 Identification of hub‐genes and risk‐score model construction

Next, we selected modules of interest in which the genes in the modules were defined to be highly correlated with certain clinical features. Next, we used univariate Cox analysis to screen for genes that were significantly associated with prognosis in the module (*P* < .001 was considered significant), and LASSO was used for further analyses. ClusterProfiler R package was used for Gene Ontology (GO) and Kyoto Encyclopedia of Genes and Genomes (KEGG) enrichment analyses of screened genes, and *P* < .05 was considered a statistically significant difference. The TCGA samples were randomly divided into two groups: 154 samples were tested internally, and 153 samples were internally verified. Expression of METTL14 and the other clinicopathological variables was not statistically significantly different between the two groups. In the internal training set (n = 154), LASSO regression was used to screen for HCC prognosis‐related genes based on lambda.min (the lambda corresponding to the smallest mean error), and the hub genes were selected. Lasso was analyzed with the "glmnet" package in R. The hub gene expression values weighted by the coefficients from the LASSO regression generated a risk score for each patient. The Survminer R package was used to find the optimal cutoff for the risk score, while receiver operating characteristic (ROC) and Kaplan‐Meier curves were used to assess the prognostic capacity of the risk scores. Finally, the Spearman's coefficients of prognostic m6A RNA methylation regulators with hub genes were calculated. Based on the expression level of METTL14, we used the tertile method to divide the samples into three groups (low, medium, and high expression) and compared the expression of each gene in the high‐expression group and the low‐expression group (*P* < .05).

### 2.5 Building and validating a predictive nomogram

A nomogram can be used to predict cancer prognosis.^17^ In the TCGA and GEO datasets, all prognostic hub genes identified by LASSO were included to build a nomogram to investigate the probability of 3‐ and 5‐year OS of patients with HCC. To assess the discrimination and accuracy of the nomogram, the concordance index (C‐index) was calculated and a calibration curve was plotted.

## 3 RESULTS

**Figure 1:**
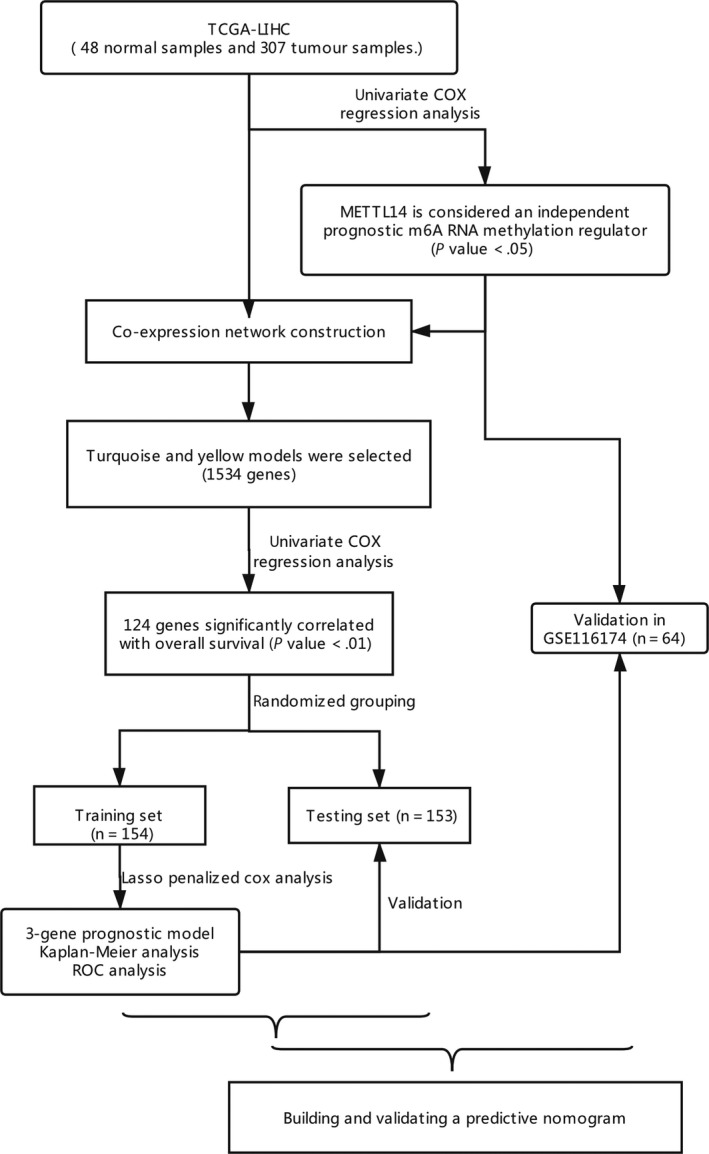
The study was conducted as described in the flow chart. TCGA, The Cancer Genome Atlas

The study was conducted as described in the flow chart (Figure 1).

### 3.1 Identification of prognostic m6A RNA methylation regulators

**Figure 2:**
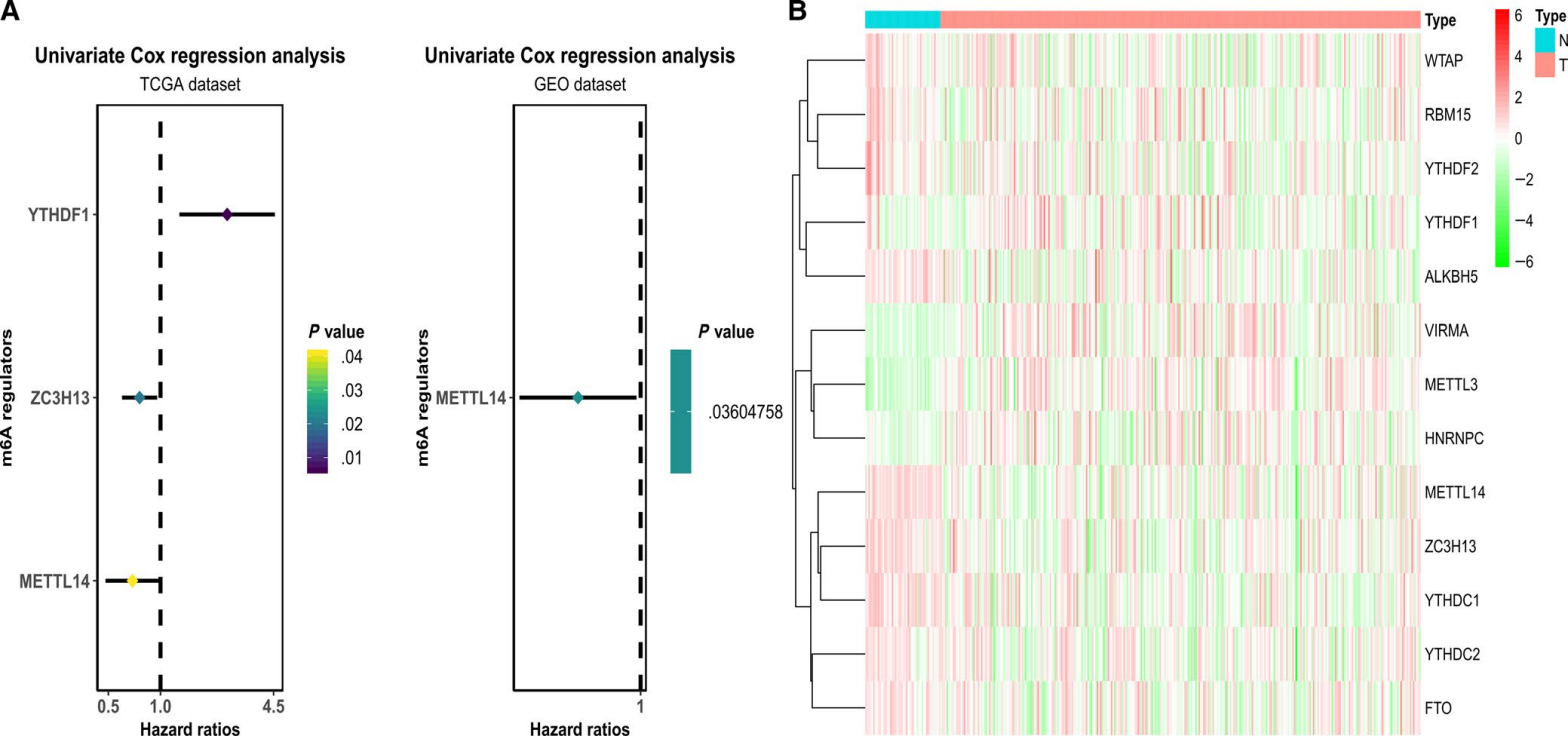
A, Forest map of univariate Cox analysis in HCC (METTL14 is associated with prognosis and is shown as a protective factor in both TCGA and http://www.ncbi.nlm.nih.gov/geo/query/acc.cgi?acc=GSE116174). B, There are significant differences in the expression levels of different genes in normal samples and tumor samples, and METTL14 in normal samples is significantly higher than that in tumor samples. GEO, Gene Expression Omnibus; HCC, hepatocellular carcinoma; N, normal tissues; T, tumor tissues; TCGA, The Cancer Genome Atlas

Univariate Cox analysis was performed to identify the m6A genes associated with the prognosis of HCC patients (a forest map was used to display the m6A regulators with *P* < .05). Based on univariate COX analysis, we found that high expression of METTL14 is associated with a better prognosis in patients with HCC. METTL14 showed *P* < .05 in the TCGA and GEO datasets, and HR < 1, which can be considered a protective factor positively affecting the prognosis of HCC patients (Figure 2A).

### 3.2 Differential expression of m6A RNA methylation regulators

In the TCGA set, 48 cases were normal samples and 307 cases were tumor samples. Heatmaps and violin maps were drawn according to the different gene expression levels. According to the results, we can conclude that METTL14 had lower expression in the tumor samples than in the normal samples. VIRMA, METTL3, and HNRNPC showed higher expression in tumors than in normal tissues, while METTL14, ZC3H13, YTHDC1, YTHDC2, and FTO showed higher expression in normal tissues (Figure 2B).

**Figure 3:**
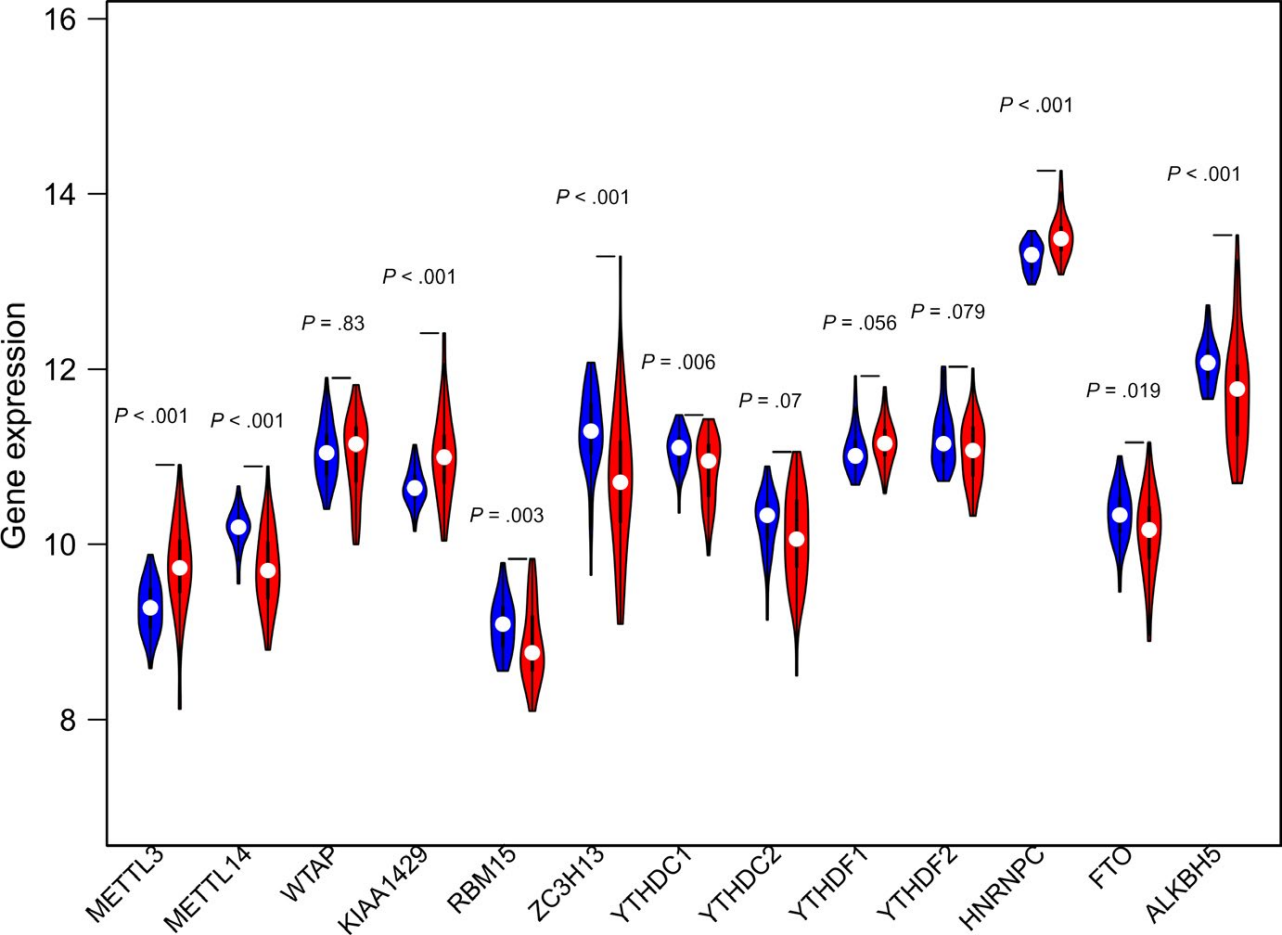
METTL14 showed higher expression in normal tissues

As shown in the violin plot (Figure 3), the expression of METTL3, METTL14, KIAA1429, RBM15, ZC3H13, YTHDC1, HNRNPC, FTO, and ALKBH5 in normal tissues is obviously different from that in tumor tissues, and the differences had significance (*P* < .05). METTL14, RBM15, ZC3H13, YTHDC1, FTO, and ALKBH5 showed higher expression in normal tissues.

### 3.3 Co‐expression network construction

**Figure 4:**
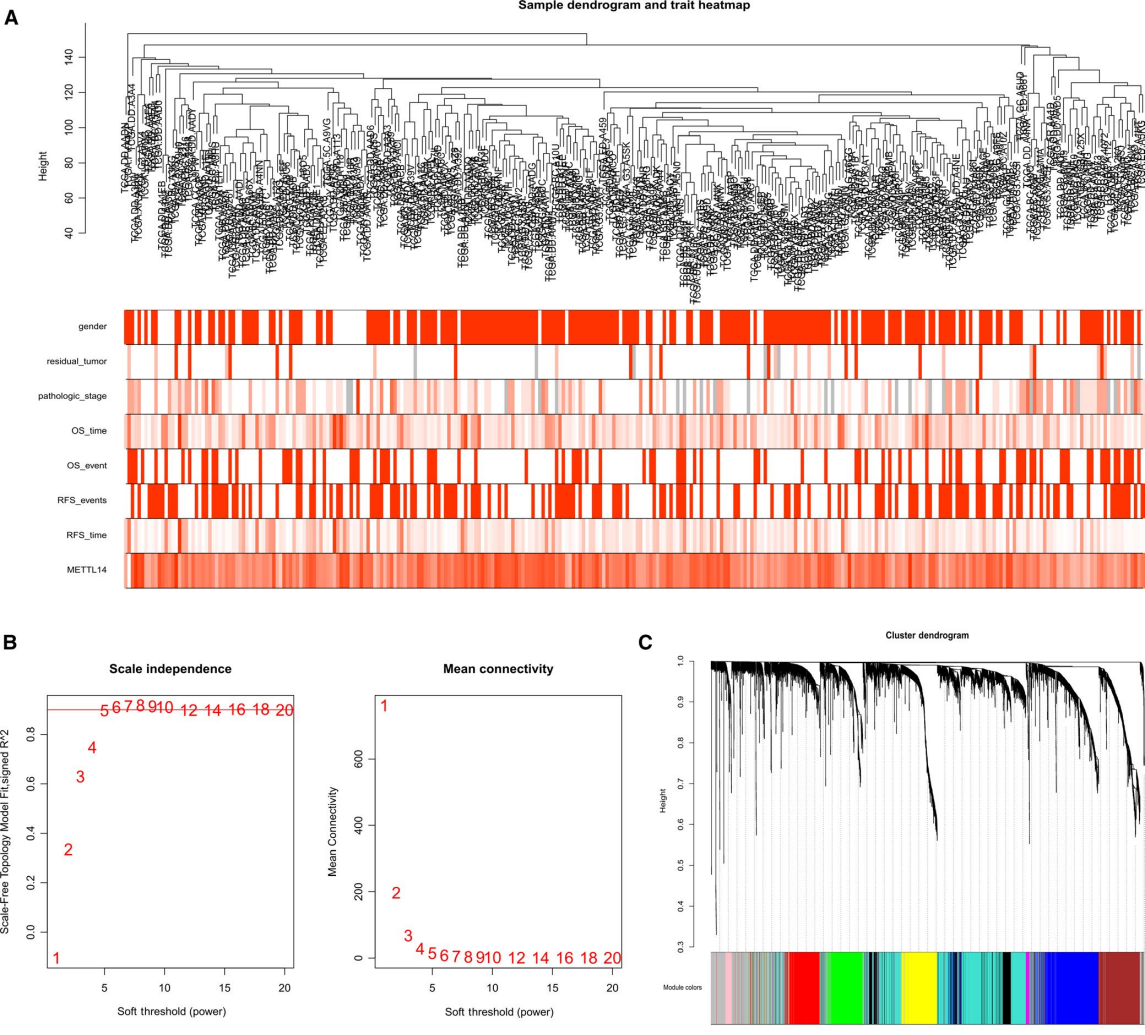
A, Clustering of 307 tumor samples and clinical information (where the number/stage is larger, the darker color is shown). B, The scale‐free index calculated under different *β* and the average connectivity calculated under different *β* (the numbers in the figure indicate the corresponding soft threshold power. The approximate scale‐free topology can be achieved at a soft threshold power of 5). C, Gene clustering tree diagram. Based on the common topological overlap, each color module represents a module that contains a set of highly connected genes

As described above, this study calculated the variance of the expression of each gene in all samples, and genes with variances greater than all variance quartiles were selected for a total of 4842 genes. After building a hierarchical clustering tree by 4842 genes in 307 tumor samples, 4 samples were considered to be outliers and were rejected. Rehierarchical clustering of the remaining 303 samples with sample clinical information (Figure 4A) was then conducted. To construct a scale‐free network, we need to choose the appropriate weighting factor *β* while moderately retaining the average connectivity of each gene node. We finally chose *β* = 5 to construct the co‐expression network (Figure 4B). After determining the *β* value, a total of 10 modules were identified (Figure 4C).

### 3.4 Correlation between modules and phenotypes

**Figure 5:**
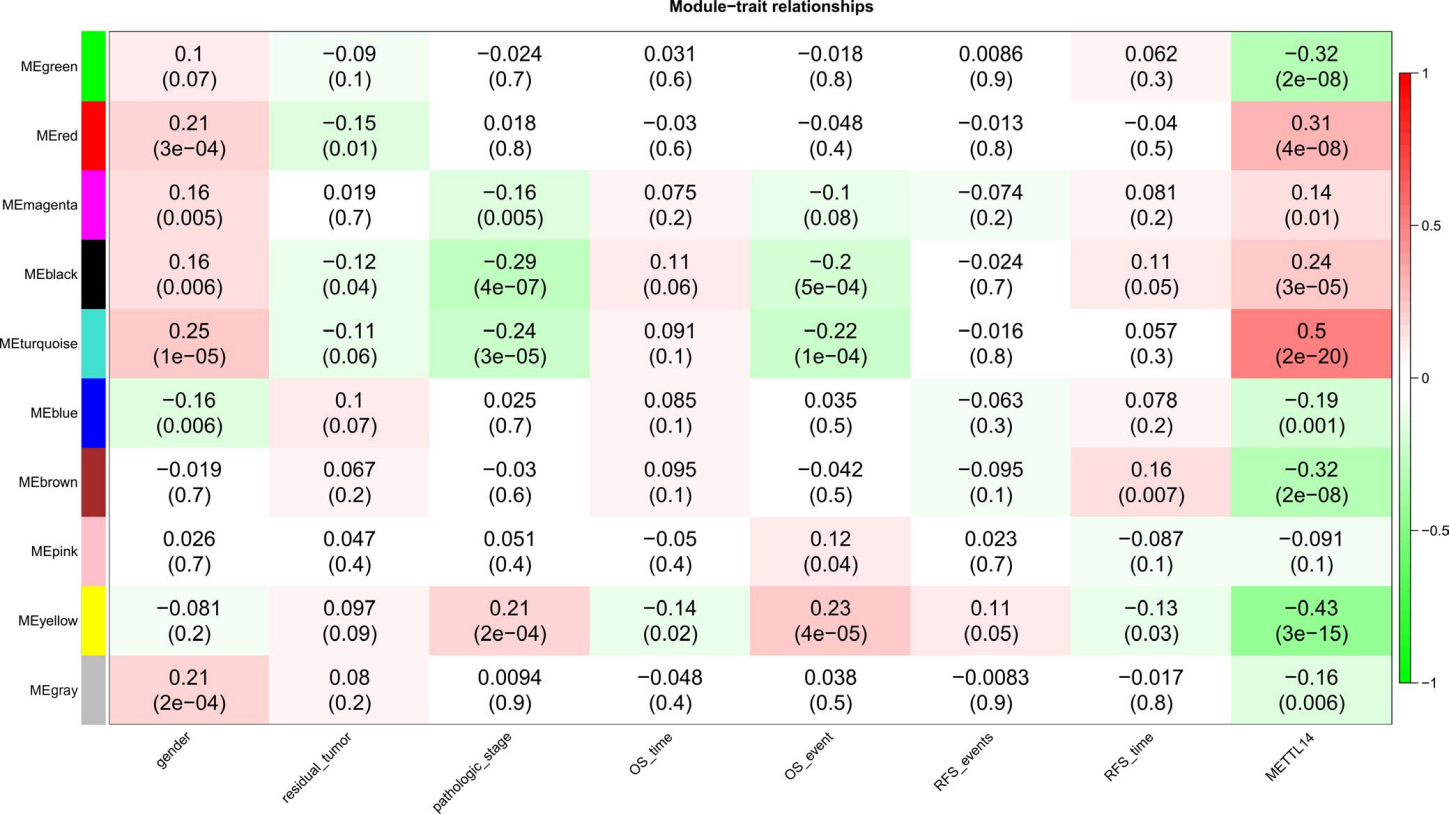
Correlation heatmap of different modules with various clinical phenotypes. Each row in the figure corresponds to a gene module, and each column corresponds to a clinical phenotype. The numbers in brackets indicate the *P* value, and the numbers without the brackets indicate the correlation

Based on the correlation of each module and clinical phenotype, modules that were significantly associated with prognosis and METTL14 expression were selected. The turquoise and yellow modules have a significantly higher correlation with METTL14 expression (positive values indicate a positive correlation, negative values indicate a negative correlation) and have a stronger correlation with the patient's OS time and pathologic stage. This suggests that the genes in the two modules may be regulated by METTL14 and play a role in the prognosis of patients with HCC (Figure 5).

### 3.5 Hub genes identification

To further identify the prognostic genes regulated by METTL14, we selected turquoise and yellow modules for the next study for a total of 1534 genes. A preliminary selection of prognostic genes was made by univariate Cox, where a *P* < .0001 was used as a cutoff for screening prognostic genes, and 124 genes were selected.

**Figure 6:**
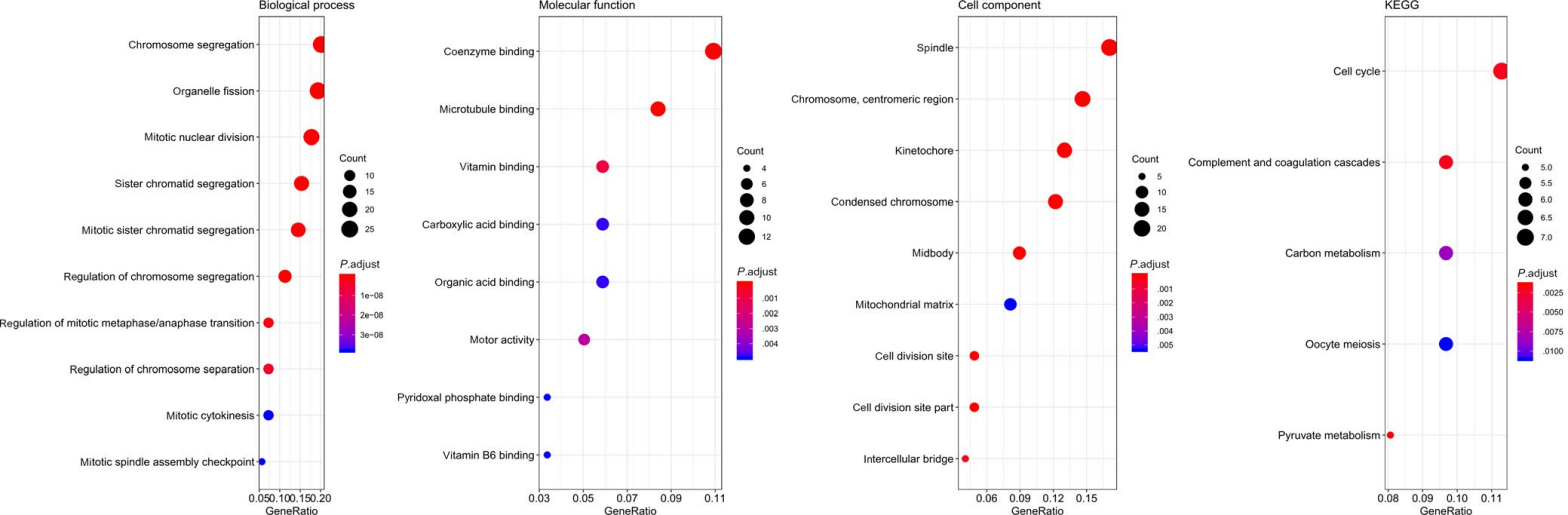
Functional enrichment analysis of 124 selected genes. KEGG, Kyoto Encyclopedia of Genes and Genomes

The 124 selected genes were analyzed by clusterProfiler R package for GO and KEGG pathway analysis. In biological process terms of GO analysis, the genes were mainly enriched in "chromosome segregation," "sister chromatid segregation," "mitotic nuclear division," "mitotic sister chromatid segregation," "organelle fission," "regulation of chromosome segregation." In cell component terms, differentially expressed genes (DEGs) were mainly enriched in "chromosome, centromeric region," "kinetochore," "spindle," "condensed chromosome," "midbody," "cell division site," "cell division site part," "intercellular bridge," "mitochondrial matrix." In molecular function terms, DEGs were mainly enriched in "coenzyme binding," "microtubule binding," "vitamin binding," "motor activity," "carboxylic acid binding," "organic acid binding," "pyridoxal phosphate binding," "vitamin B6 binding." KEGG pathway analysis demonstrated that 124 selected genes were significantly enriched in "Pyruvate metabolism," "Cell cycle," "Complement and coagulation cascades," "Carbon metabolism," "Oocyte meiosis," "Tyrosine metabolism," "Fatty acid degradation," "Valine, leucine and isoleucine degradation," "Cysteine and methionine metabolism," "Progesterone‐mediated oocyte maturation" and "Phenylalanine metabolism," etc (Figure 6).

Then, 307 TCGA samples were randomly divided into an internal training set and an internal testing set. The tableone R package was used to describe the clinical information difference between the internal training set and the internal testing set. The results showed that expression of METTL14 and the other clinicopathological variables was not significantly different between the two groups (Table 1). The 64 samples of the http://www.ncbi.nlm.nih.gov/geo/query/acc.cgi?acc=GSE116174 were treated as the external testing set.

**Table 1:** There was no significant difference between the two groups of clinical phenotypes

	Training set	Testing set	*P* value
n	154	153	
Gender = male (%)	105 (68.2)	104 (68.0)	1
residual_tumor (%)			.121
R0	140 (92.1)	130 (87.2)	
R1	7 (4.6)	7 (4.7)	
R2	1 (0.7)	0 (0.0)	
RX	4 (2.6)	12 (8.1)	
Pathologic_stage (%)			.68
Stage I	71 (50.0)	71 (49.7)	
Stage II	36 (25.4)	29 (20.3)	
Stage III	2 (1.4)	1 (0.7)	
Stage IIIA	26 (18.3)	30 (21.0)	
Stage IIIB	3 (2.1)	4 (2.8)	
Stage IIIC	3 (2.1)	6 (4.2)	
Stage IV	1 (0.7)	0 (0.0)	
Stage IVB	0 (0.0)	2 (1.4)	
OS_time (median [IQR])	640.50 [391.25, 1228.00]	615.00 [344.00, 1091.00]	.442
OS_event = 0/1 (%)	111/43 (72.1/27.9)	100/53 (65.4/34.6)	.252
RFS_events = 0/1 (%)	77/77 (50.0/50.0)	86/67 (56.2/43.8)	.329
RFS_time (median [IQR])	420.00 [184.00, 747.75]	393.00 [201.00, 816.00]	.956
METTL14 (mean (SD))	9.76 (0.54)	9.68 (0.46)	.174

Abbreviations: OS, overall survival; RFS, relapse free survival.

**Figure 7:**
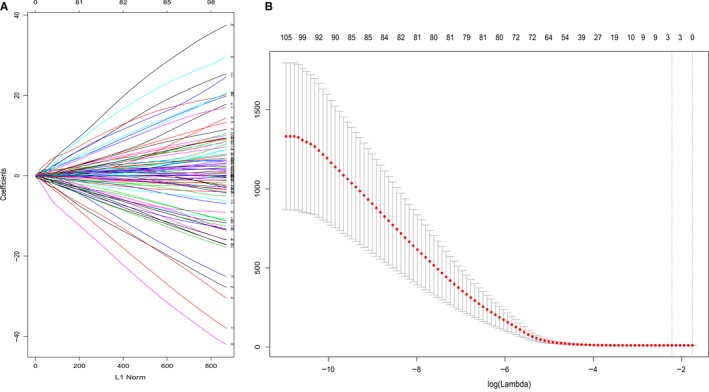
A, Distribution of least absolute shrinkage and selection operator (LASSO) coefficients for 124 genes. B, Partial likelihood deviation of the LASSO coefficient distribution. Vertical dashed lines indicate lambda.min and lambda.1se

In the internal experimental group, a total of 124 prognostic genes were screened for the two modules using LASSO analysis (optimal lambda value 0.1086875). The results showed that cysteine sulfinic acid decarboxylase (CSAD), glutamic‐oxaloacetic transaminase 2 (GOT2), and suppressor of cytokine signaling 2 (SOCS2) are real hub genes that are associated with patient prognosis (Figure 7).

**Figure 8:**
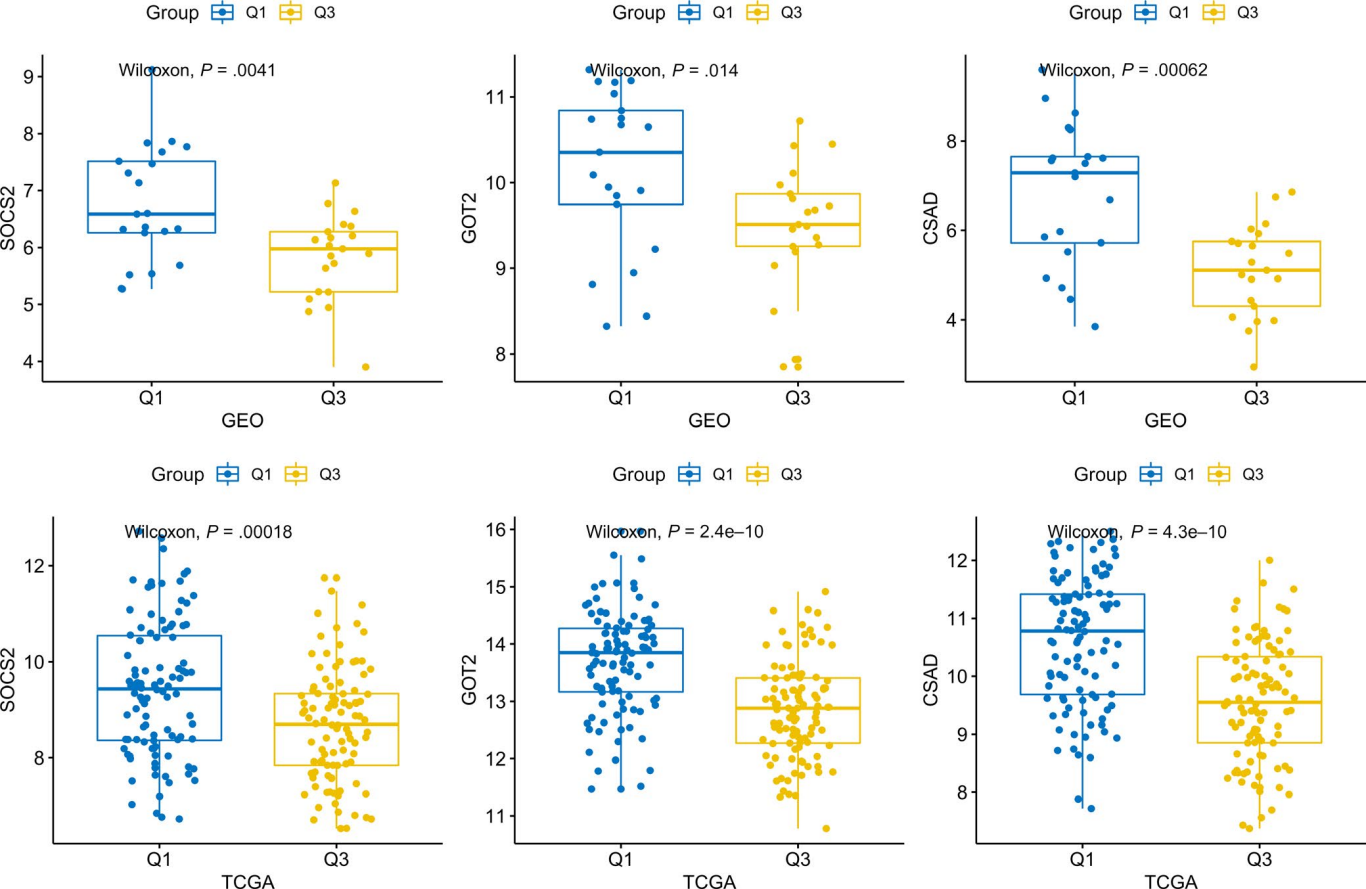
Differences in hub gene expression among high, moderate, and low METTL14 expression groups

**Figure 9:**
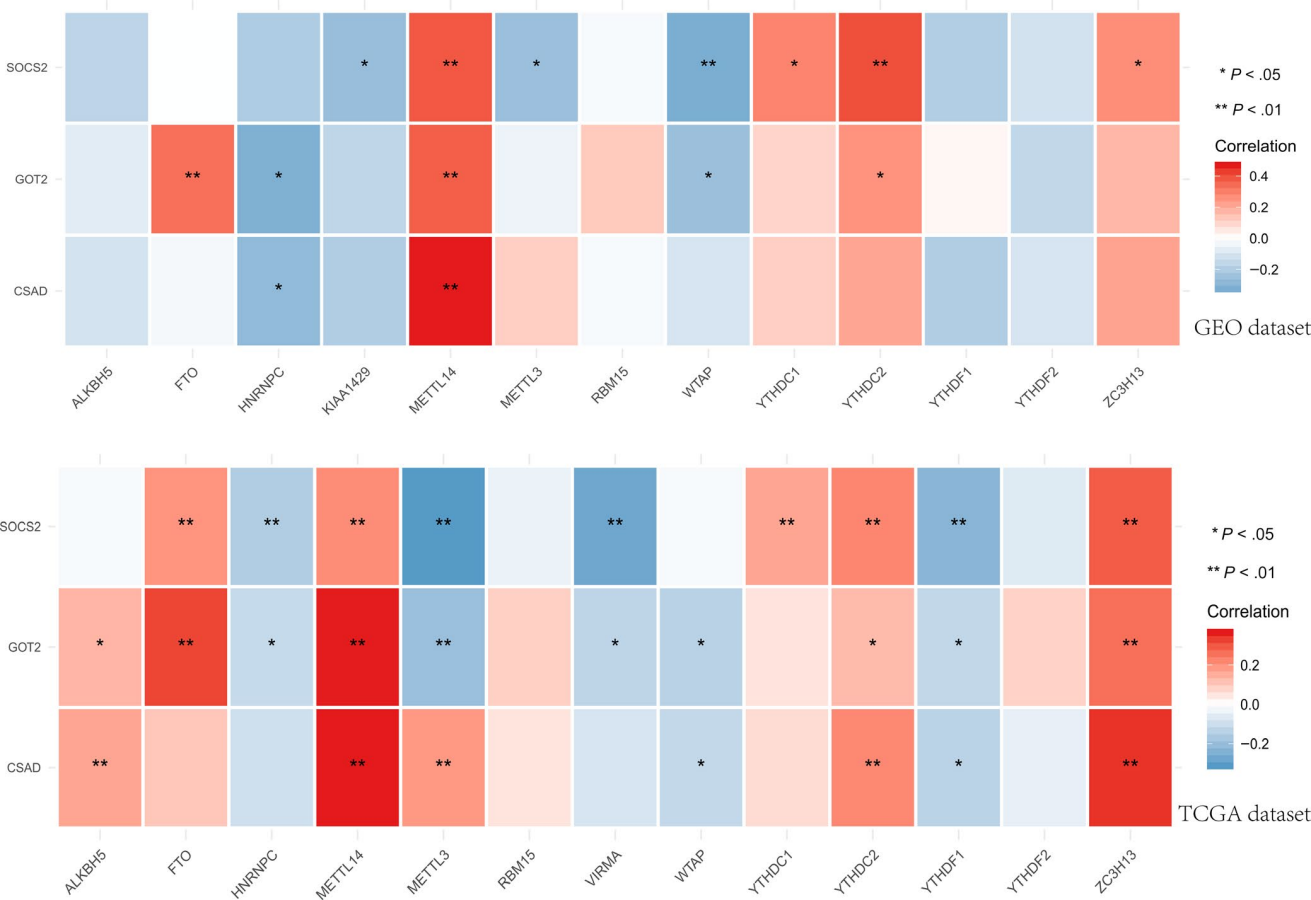
Expression of METTL14 and hub genes showed a significant correlation. GEO, Gene Expression Omnibus; TCGA, The Cancer Genome Atlas

Based on the expression level of METTL14, we used the tripartite method to divide the samples into three groups (low, medium, and high expression) and compared the expression of hub genes in the two groups with high expression in Q1 and low expression in Q3. The results showed (Figure 8) the following: In the low‐expression and high‐expression groups of METTL14, the expression of hub genes was significantly different; in the Q1 group with high expression of METTL14, the hub genes were often highly expressed; and in the Q3 group with low expression of METTL14, they often had low expression. In both the TCGA and GEO datasets, a significant correlation was found between the expression of METTL14 and that of the hub genes (*P* < .05) (Figure 9).

### 3.6 Risk scores

Three genes were identified and subsequently used to construct a prognostic gene signature. The risk score = −(0.17877930 × CSAD + 0.10997478 × GOT2 + 0.04458126 × SOCS2), and we used the Survminer R package to find the optimal cutoff for the risk score, while ROC and Kaplan‐Meier curves were used to assess the prognostic ability of the risk scores.

**Figure 10:**
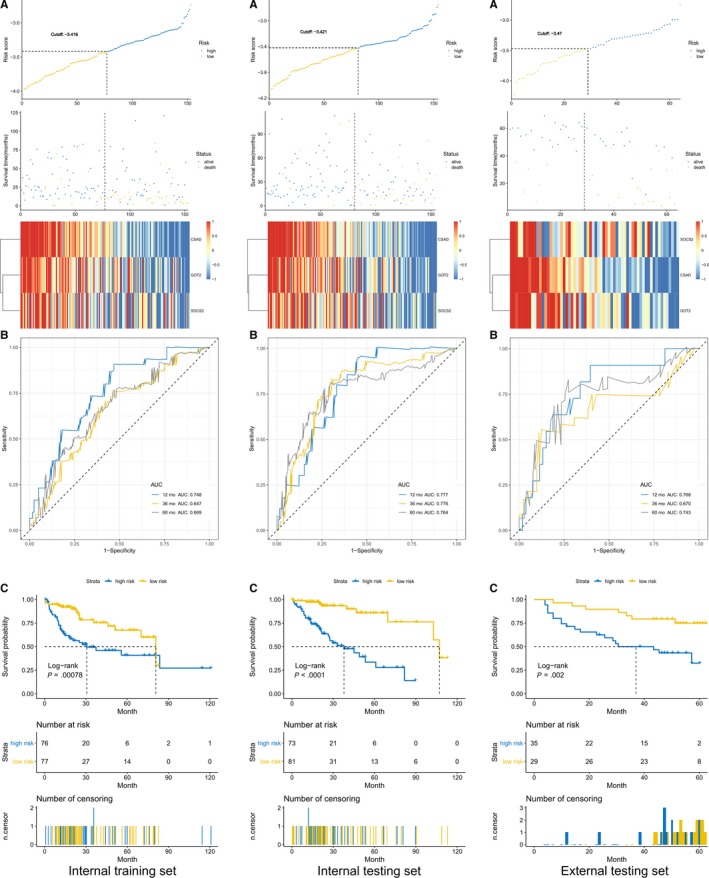
Risk score, heatmap of mRNA expression, time‐dependent ROC analysis, and Kaplan‐Meier curve of the 3‐gene signature in the internal training set, internal testing set, and external testing set. A, Risk score, heatmap of mRNA expression, B, Time‐dependent ROC analysis, and C, Kaplan‐Meier curve of the 3‐gene signature. AUC, area under the ROC curves; HCC, hepatocellular carcinoma; ROC, receiver operating characteristic

We plotted the risk score distribution, the time‐dependent ROC curve and the survival analysis of the internal training set, internal testing set, and external testing set (Figure 10). The area under the ROC curves (AUCs) of the OS prognostic model were as follows: 12 month AUC: 0.748, 36 month AUC: 0.647, 60 month AUC: 0.669; 12 month AUC: 0.777, 36 month AUC: 0.776, 60 month AUC: 0.764; and 12 month AUC: 0.768, 36 month AUC: 0.670, 60 month AUC: 0.743. Collectively, our results indicated a good performance of the three‐gene signature for survival prediction.

### 3.7 Build a nomogram based on the hub genes

**Figure 11:**
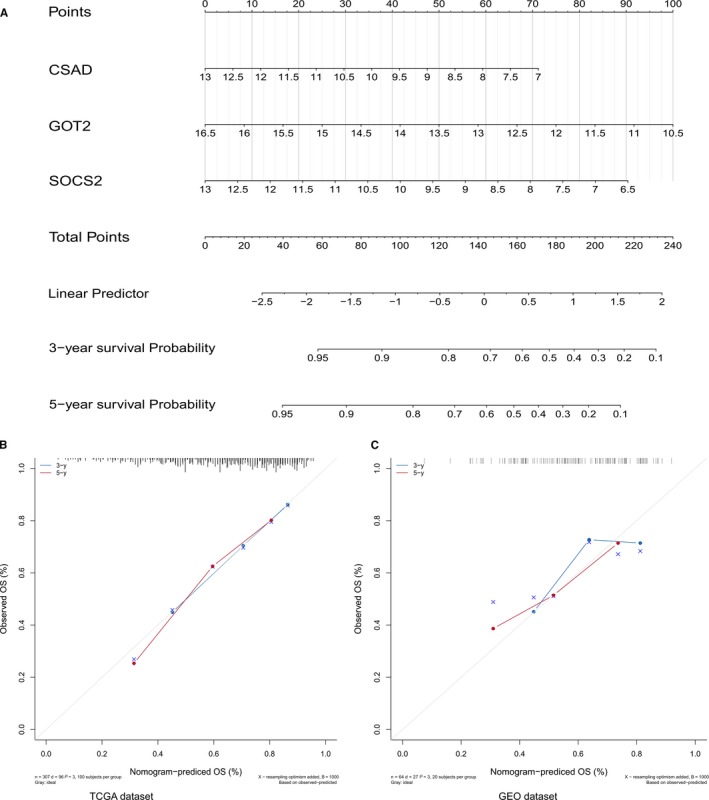
A, Nomogram predicting OS for HCC patients. B and C, 3‐ and 5‐y survival calibration curves of the TCGA dataset and GEO dataset. The 3‐y survival probability curve is the blue line, the 5‐y survival probability curve is the red line, and the ideal curve is gray. GEO, Gene Expression Omnibus; HCC, hepatocellular carcinoma; OS, overall survival; TCGA, The Cancer Genome Atlas

For the prediction of 3‐ and 5‐year OS, we built a nomogram (Figure 11A). Calibration curves and the C‐index were used to assess the discrimination and accuracy of the nomogram. In the TCGA dataset, the C‐index was 0.7307009, and in the GEO dataset, the C‐index was 0.67633229. The 3‐ and 5‐year survival probability calibration curves for the TCGA and GEO datasets show that the calibration curve is close to the ideal curve, indicating that the nomogram has good predictive effects (Figure 11B,C).

## 4 DISCUSSION

In this study, we first evaluated the expression of METTL14 in HCC, and the expression of METTL14 in tumor samples was found to be significantly reduced, which is consistent with the findings of a previous study. METTL14 can positively regulate the primary microRNA 126 process in an m6A‐dependent manner to inhibit the metastatic potential of HCC.^12^ METTL14, as an important m6A methyltransferase, has been identified as playing an important role in many physiological functions^18^, ^19^, ^20^, ^21^ and is also related to the occurrence and development of a variety of cancers.^12^, ^22^, ^23^ In addition, METTL14 has become a new target for treatment in EBV‐associated tumors.^24^

We finally identified three hub genes (CSAD, GOT2, and SOCS2) that may be regulated by METTL14. In previous studies, these three genes have been found to be involved in the development of many diseases, such as retinal abnormalities.^25^, ^26^, ^27^ CSAD maintains its high expression when stimulated in the precancerous liver,^28^ and gene polymorphisms of GOT2 are closely related to occupational exposure to liver injury,^29^ and in particular, GOT2 was found to be involved in many cancers, such as pancreatic tumors.^30^ Hong et al reported that BRCA1 modulates aspartate biosynthesis through transcriptional repression of GOT2,^31^ while Maren Feist et al reported that GOT2 is a biomarker of lymphoma.^32^ Similarly, many studies have shown that SOCS2 plays an important role in inhibiting the progression of liver cancer.^33^, ^34^, ^35^ Previous studies have shown that our three hub genes play a large role in the development of tumors, and our study has further confirmed the relationship among these three hub genes and the m6A RNA methylation regulator.

The authors of a previous study have suggested that METTL3 could repress the expression of SOCS2 in HCC by an m6A‐YTHDF2‐dependent mechanism^34^ and that KIAA1429 plays a role in regulating ID2 expression by regulating m6A of ID2 mRNA.^36^ Similarly, METTL14 may participate in the malignant progression of HCC by adjusting the m6A of CSAD, GOT2, and SOCS2; however, this remains to be verified by further experiments. To our knowledge, this three‐gene signature and nomogram have been proposed by us for the first time, and it might be useful for prognostication and diagnosis of HCC.

## 5 CONCLUSION

In this study, we found that METTL14 may inhibit the progression of HCC by upregulating the expression levels of CSAD, GOT2, and SOCS2. The main mechanism might be via affecting the m6A process of the hub genes. In addition, we established a novel three‐gene signature and nomogram to predict OS of HCC, which might be a useful prognostic and diagnostic classification tool of HCC.

## CONFLICT OF INTEREST

The authors have no conflicts of interest to declare.

## ETHICAL STATEMENT

The data of this study are from TCGA, GEO, and GTEx database, and do not involve animal experiments and human specimens, and no ethics‐related issues.

## CONSENT FOR PUBLICATION

The authors listed have approved the manuscript that is enclosed.

## Data Availability

The data of this study are from TCGA and GEO database.
